# Improving structural and magnetic properties of zinc stannate thin films through nickel doping via sol–gel method

**DOI:** 10.1038/s41598-024-63209-2

**Published:** 2024-06-26

**Authors:** Ibrahim Cinar

**Affiliations:** Department of Medical Services and Techniques, Karamanoglu Mehmetbey University, 70100 Karaman, Turkey

**Keywords:** Ternary metal oxide material, Sol–gel technique, Zinc stannate, Structural analysis, Magnetic properties, Solar cells, Photonic devices

## Abstract

Ternary oxides are currently emerging as promising materials for optoelectronic devices and spintronics, surpassing binary oxides in terms of their superior properties. Among these, zinc stannate (Zn_2_SnO_4_) stands out due to its stability and attractive physical characteristics. However, despite its outstanding attributes, there is a need to further develop its magnetic properties for spintronic applications. In this study, Ni-doped Zn_2_SnO_4_ thin films were synthesized using the sol–gel method, and their magnetic characteristics were investigated for the first time. X-ray diffraction analysis confirmed the high crystallinity of the synthesized samples, even after the incorporation of Ni dopants, without any secondary phases. SEM imaging revealed the cubic structure morphology of the thin films. An increase in the bandgap, dependent on the Ni dopant concentration, was observed for doped zinc stannate, suggesting potential for tailored electronic properties. FTIR spectroscopy confirmed the presence of functional groups within the material. Notably, the magnetic properties of the thin films were analyzed using a vibrating sample magnetometer (VSM), revealing diamagnetic behavior for pure zinc stannate and ferromagnetic properties for Ni-doped Zn_2_SnO_4_, which increased with dopant concentration. Overall, the results highlight the excellent structural, optical, and ferromagnetic properties of Ni-doped Zn_2_SnO_4_ thin films, positioning them for diverse applications, particularly in optoelectronic and spintronic technology.

## Introduction

Recent researches emphasize the significance of nanotechnology in electronics and magnetism, and the growing interest in transition metal oxides nanoparticles by highlighting the potential of combining electronics and ferromagnetism for various applications^1–8^.

Transition metal-doped semiconductor oxides (TMO) have been explored for their ability to generate carrier-mediated ferromagnetism^9–12^. Room temperature ferromagnetism in these materials’ results from intrinsic defects, impurity phases, or ferromagnetic precipitates rather than the magnetic moments of transition metal ions. Ternary oxides, in particular, offer improved properties compared to binary oxides and can be tailored for applications like optoelectronic devices and spintronics^13,14^ . While room temperature ferromagnetism has been observed in transition metal-doped binary semiconductor oxides like ZnO and SnO_2_^15–21^, there's limited research on ternary oxide Zinc stannate (ZnSnO_3_ and Zn_2_SnO_4_), known as ZTO, doped with transition metal ions^22^.

ZTO is another important material known for its multifunctional properties because of a stable compound within the ZnO–SnO_2_ system and serves as a crucial TMO. It finds extensive use in optoelectronic devices like solar cells, liquid crystal displays, energy-efficient windows, and invisible electronic circuits^23,24^. ZTO crystallizes in a face-centered cubic spinel structure with a wide band gap of.

3.6 eV and high electron mobility, making it suitable for many applications^24–28^. Moreover, ZTO exhibits multifunctional properties, including low electron–hole recombination rates and longer electron lifetimes, making it ideal for opto-/micro-electronic technology^1,22,25,26,29,30^. It also demonstrates efficient photocatalytic activity, especially for degrading organic dyes in aqueous.

solutions due to its high charge separation induced by surface oxygen vacancies^31–33^.

However, despite their outstanding characteristics, their diamagnetic nature poses a problem for spintronic applications, yet various methods are being used to enhance their magnetic properties. Doping 3d transition-metal ions into semiconductors can dramatically alter their electrical structure and lead to fascinating multi-functionality. Transition metal (TM) doping, such as Mn^2+^, Fe^2+^ , Co^2+^ and N^2+^ influences the electronic structure of semiconductors^30,34–37^. While room-temperature ferromagnetism has been extensively studied in TM-doped binary semiconductor oxides like ZnO and SnO_2_, there is limited research on magnetic properties in ternary systems like ZTO doped with TM ions^34,38^. Co-doping with Co, in particular, is effective in manipulating magnetism through interactions with vacancies and defects in TM-doped diluted magnetic semiconductors, making it a valuable tool for exploring room-temperature ferromagnetism. However, their presence also leads to the formation of defects that alter the electronic structure and contribute to observed magnetism. Incorporating magnetic dopants not only introduces d-electron magnetism but also increases the content of oxygen vacancies, thus affecting the material's properties. Understanding the origin of room-temperature ferromagnetism in non-magnetic ternary oxides like ZTO is critical for harnessing their potential in various technological applications. Also, synthesis methods play a crucial role in determining the crystal structure and stability of the materials, which, in turn, affects their properties, with various methods being explored to control morphology and composition.

Herein, Ni-doped ZTO thin films were effectively synthesized by sol–gel process, and the influence of doping concentration on the structural, optical, and magnetic characteristic of the these thin film was explored. More importantly, ZTO thin films have been proven to effectively acquire ferromagnetic characteristics by the incorporation of nickel into the host matrix. These findings suggested that Ni-doped ZTO thin films might be a potential ferromagnetic material for developing highly efficient spintronic devices.

## Experimental

All sample fabrication processes were conducted under identical conditions and simultaneously. We utilized high-purity reagent-grade chemicals without further purification in the synthesis of nanoparticles (NPs). We modified the following study of Shin at all in 2015^29^. In the preparation of ZTO NPs, 12.8 mmol of ZnCl_2_ (sourced from Aldrich) and 6.4 mmol of SnCl_4_ × 5H_2_O (also from Aldrich) were dissolved in 160 ml of deionized water under vigorous magnetic stirring. Following this, N_2_H_4_ x H_2_O, with a molar ratio of N_2_H_4_ to Zn of 8:1, was introduced into the reaction solution. For the synthesis of nickel-doped ZTO, the same procedure was followed with adding Ni(NO_3_)_2_ × 5H_2_O (in varying ratios of 1%, 2%, 3%, 5%, and 10%). White precipitates formed immediately in both cases, and these solutions, including the precipitates, were heated on a hot plate at 100 °C for a period of 12 h. The products underwent thorough washing with deionized water and ethanol before being dispersed in 2-methoxy ethanol to form a colloidal solution. Subsequently, the ZTO powder obtained was annealed up to 400 °C to facilitate ZTO crystallization.

For the fabrication of thin films ZTO on silicon (Si) wafer substrates, the substrates underwent the Radio Corporation of America (RCA) cleaning procedure followed by exposure to UV ozone for 30 min to prevent contamination and enhance nanoparticle attachment to the surface^39^. Subsequently, a ZTO thin film was formed by spin-coating 40 ml of the colloidal ZTO particle dispersion onto the Si wafer substrate at 3000 rpm for 30 s. The coated substrates were then dried on a hot plate at 100 °C for 3 min. This spin-coating and drying process was repeated 30 times to achieve the desired thin film of ZTO on the Si wafer substrate.

“The structural and phase identification of the thin films were characterized using the GI-X-ray diffraction (XRD) method, employing a Bruker D8 Advanced X-ray diffractometer with Cu Kα1 radiation (λ = 1.5406 Å). Fourier-transform infrared (FT-IR) spectra were obtained using a BRUKER VERTEX 70 Perkin Elmer FT-IR spectrophotometer. Surface morphology was analyzed with a HITACHI SU5000 field emission-scanning electron microscope (FE-SEM). Magnetic parameters were determined utilizing the on a DynaCool-9 Quantum Design physical properties measurement system (PPMS) Vibrating Sample Magnetometer (VSM). Optical properties were studied using a Shimadzu 2600 UV–Visible spectrometer.

## Results and discussion

**Figure 1:**
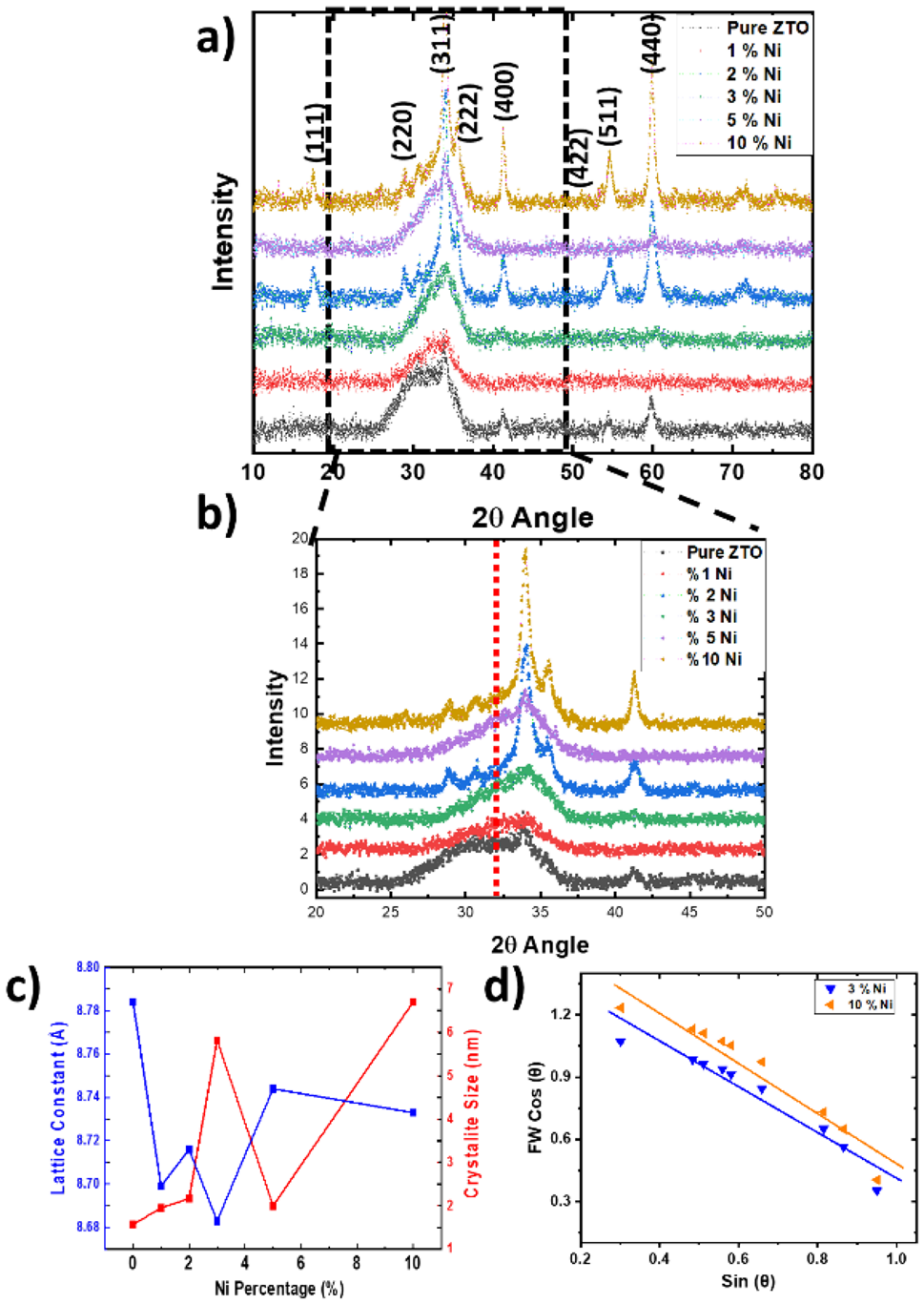
(**a,b**) XRD patterns of pure and Ni-doped ZTO thin films, (**c**) lattice constant and crystallite size vs Ni percentage, (**d**) Williamson–Hall analysis for 3% and 10%.

To explore the impact of Ni doping concentration on the crystalline structure of the host material, X-ray diffraction patterns of the prepared samples were initially recorded within the range of 2θ = 10°–80°. The XRD pattern of the prepared Ni-doped ZTO thin films is depicted in Fig. 1. All XRD (111), (220), (311), (222), (400), (511), and (440) planes patterns align with the standard diffraction pattern of the cubic inverse spinel phase of ZTO (JCPDS card No. 00-024-1470)^40^. The XRD results indicate that all thin films exhibit a single phase without any detectable secondary phases, suggesting successful incorporation of Ni atoms into the ZTO lattice. The results from the XRD analysis display irregularities, indicating potential inconsistencies in the sample structure. However, for 3% and 10% Ni-doped ZTO has an overmatch with JCPDS card more.

Furthermore, Fig. 1 illustrates that the position of the diffraction peaks of the Ni-doped ZTO thin films slightly shifts to higher angles relative to pure ZTO This shift is attributed to the smaller ionic radius of Ni^+2^ (0.69 Å) compared to Zn^+2^ (0.74 Å)^41^. The replacement of smaller Ni^+2^ ions for larger Zn^+2^ in zinc stannate also results in a decrease in lattice constants.

The average crystallite size of the synthesized sample along the most intense peak was calculated from the line broadening of the XRD diffraction peaks using the Debye–Scherrer equation^2^.

$$D=\frac{K\lambda }{\beta Cos\theta }$$

where β represents the full width at half maximum (FWHM), λ denotes the wavelength of the incident X-ray (1.5406 Å), θ stands for the angle at which the maximum peak occurs, and K is the shape factor, typically with a value of approximately 0.89^42^.

$$a=\frac{d}{\sqrt{({h}^{2}+{k}^{2}+{l}^{2})}}$$

where d is inter planar spacing for the planes indicated by Miller indices (hkl)^42^. The lattice constants of the pure and Ni-doped ZTO thin film, are illustrated in Fig. 1c). Interestingly, there appears to be no correlation between the increase in Ni doping concentration from 0 to 10% and the average crystallite size, which varies between 1.95 and 6.70 nm. This implies that the incorporation of Nickel into the zinc stannate lattice impedes the growth of zinc stannate crystallites. Additionally, a Williamson-Hall analysis was conducted to assess the strain behavior (in Fig. 1d)). A positive slope value signifies the generation of tensile strain, while a negative slope value indicates the presence of compressive strain^42,43^. In our observations, we noted a negative slope on the graph, indicating compression in the structure. This incremental trend primarily arises from the heightened structural distortion resulting from the incorporation of Ni dopants into ZTO.

**Figure 2:**
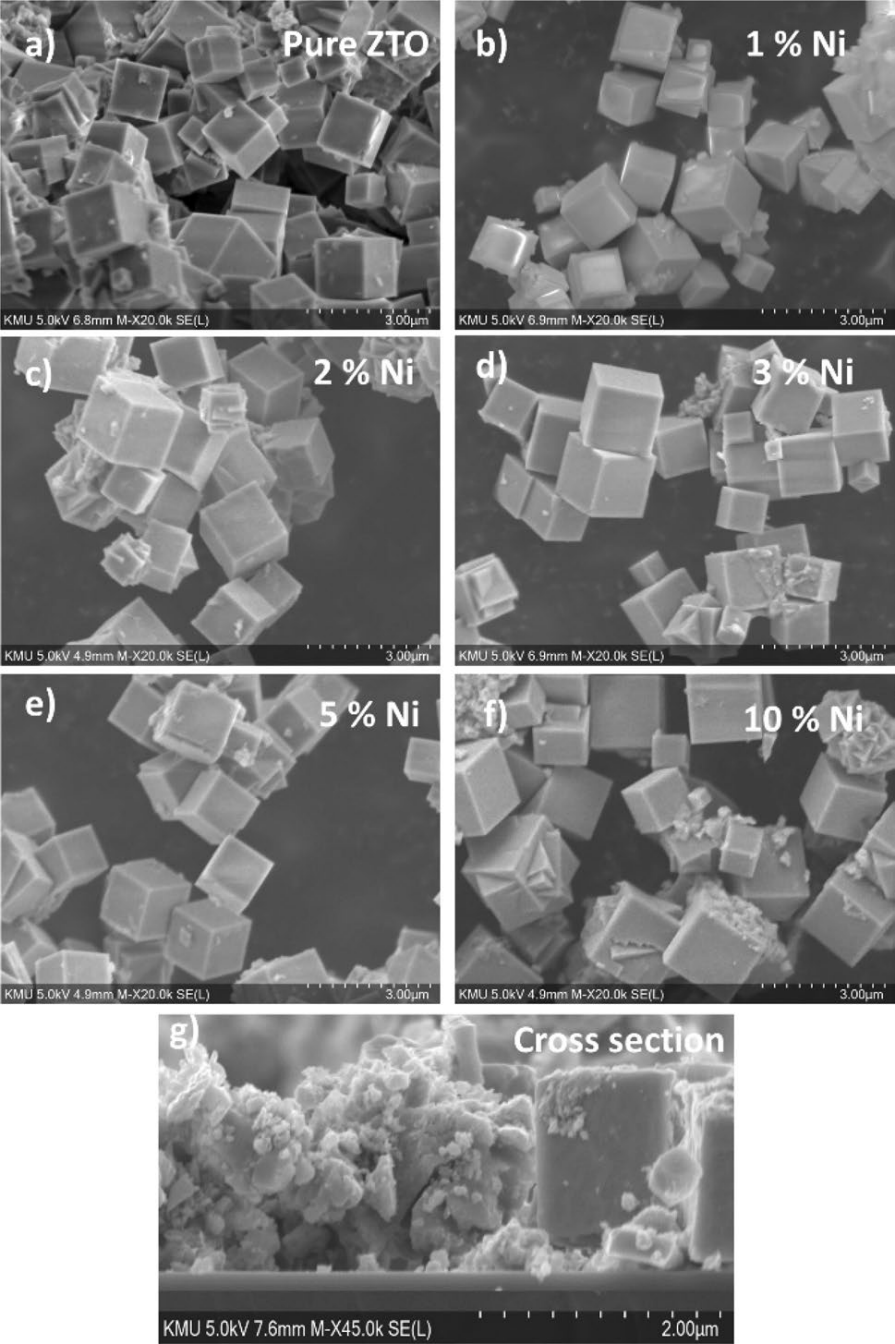
SEM images of pure and Ni-doped ZTO (**a**) pure, (**b**) 1%, (**c**) 2%, (**d**) 3%, (**e**) 5% and (**f**) 10% Ni. (**g**) Thin film cross section of Pure ZTO.

SEM analysis was employed to study the surface morphology of both pure and Ni-doped ZTO powder and cross section of thin film, with corresponding images presented in Fig. 2a–g. The SEM micrographs reveal a well-defined cubic distribution of particles in all samples, showcasing a predominantly clear surface morphology. These images support the notion of cubic structure, approximately transitioning from a scale of 0.5 to 1.7 µm (measured in SEM system), with an observed increase in general particle size, agreement with literature^22,44,45^. The presence of Nickel dopants in zinc stannate contributes to the observed increase in particle size. Furthermore, particle aggregation is evident from the images.

**Figure 3:**
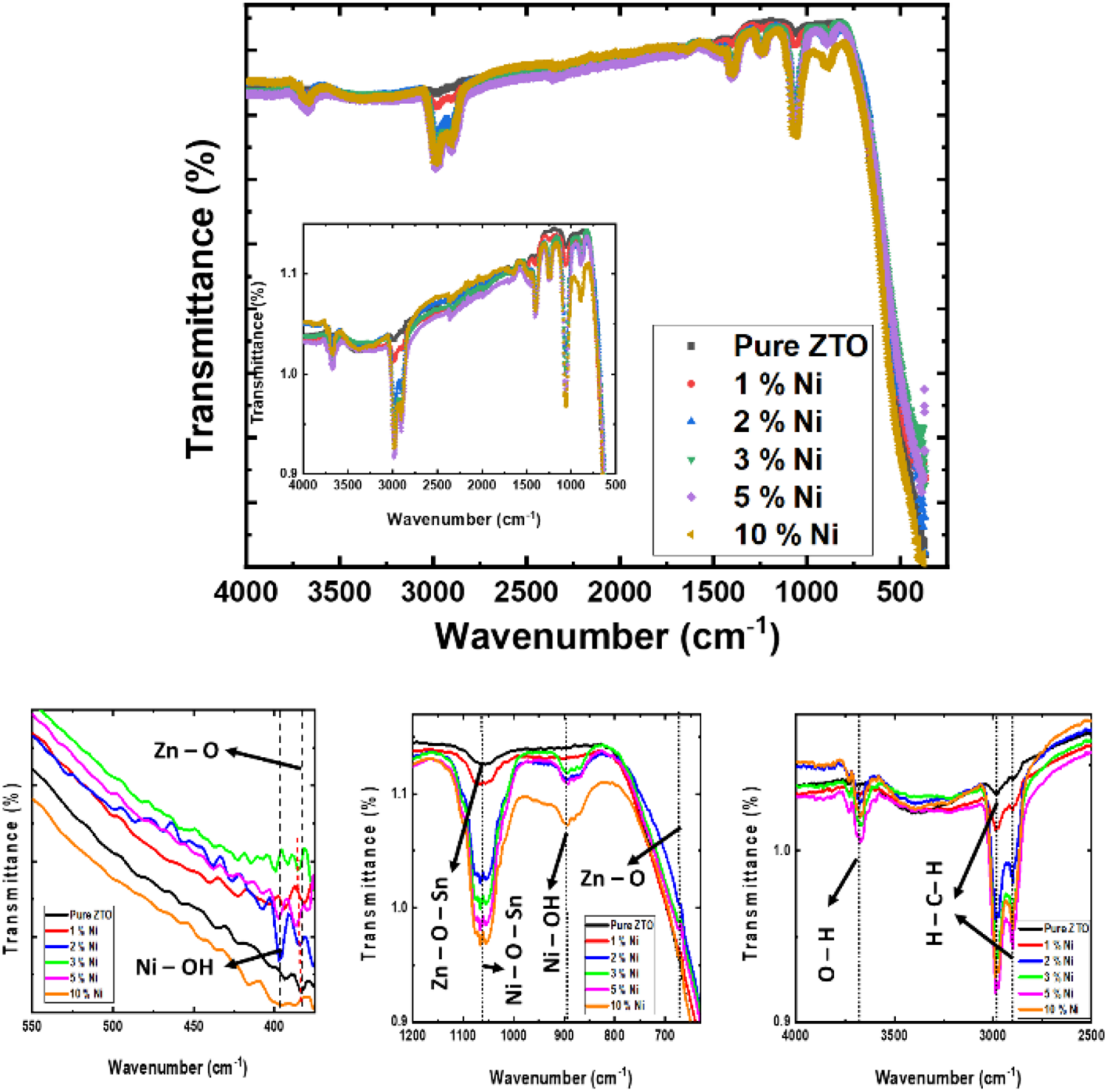
Fourier transform infrared (FTIR) spectrums of pure and Ni-doped ZTO. The inset illustrates upper side. FTIR spectrum ranges (550–380 cm^−1^), (1200–670 cm^−1^) and (4000–2500 cm^−1^) for sub-figures.

To determine the primary functional groups present in the catalyst, FTIR spectra were recorded at room temperature for both pure and Ni-doped ZTO thin films, as depicted in Fig. 3. In the FTIR spectra, peaks at approximately 397 cm^−1^ and 895 cm^−1^ are attributed to the metal–oxygen-hydrogen bending vibration of Zn–O and Ni–O in this system^46,47^. Also there is an band at 671 cm^−1^ corresponding to vibration of Zn–O^46^. Two prominent bands at around 1226 cm^−1^ and 1390 cm^−1^ were identified as the asymmetric and symmetric stretching modes of coordinated vibration modes of νs (C–O) and δ (OC=O)^48,49^. The broadness of certain peaks suggests that the Ni–O catalyst exhibits a crystalline nature. The absorption band at 2354 cm^−1^corresponds to the symmetric and asymmetric stretching modes of vibrations of the CO_2_ molecule absorbed from the air^47^. Furthermore, peaks in the region of 2894–2982 cm^−1^ are indicative of stretching vibrations of aliphatic C–H structures that remain intact^47^. Additionally, various peaks were observed, such as those between 400–1100 cm^−1^ for Ni–O bonds and 3000–3600 cm^−1^ from vibration mode of the hydrogen bond hydroxyl group^50^. Based on the FT-IR results, it is evident that the Ni-doped ZTO thin films exhibits sharp characteristic peaks, indicating the high crystalline nature of Ni-doped ZTO depending on the amount of Ni dopants (Fig. 3).

**Figure 4:**
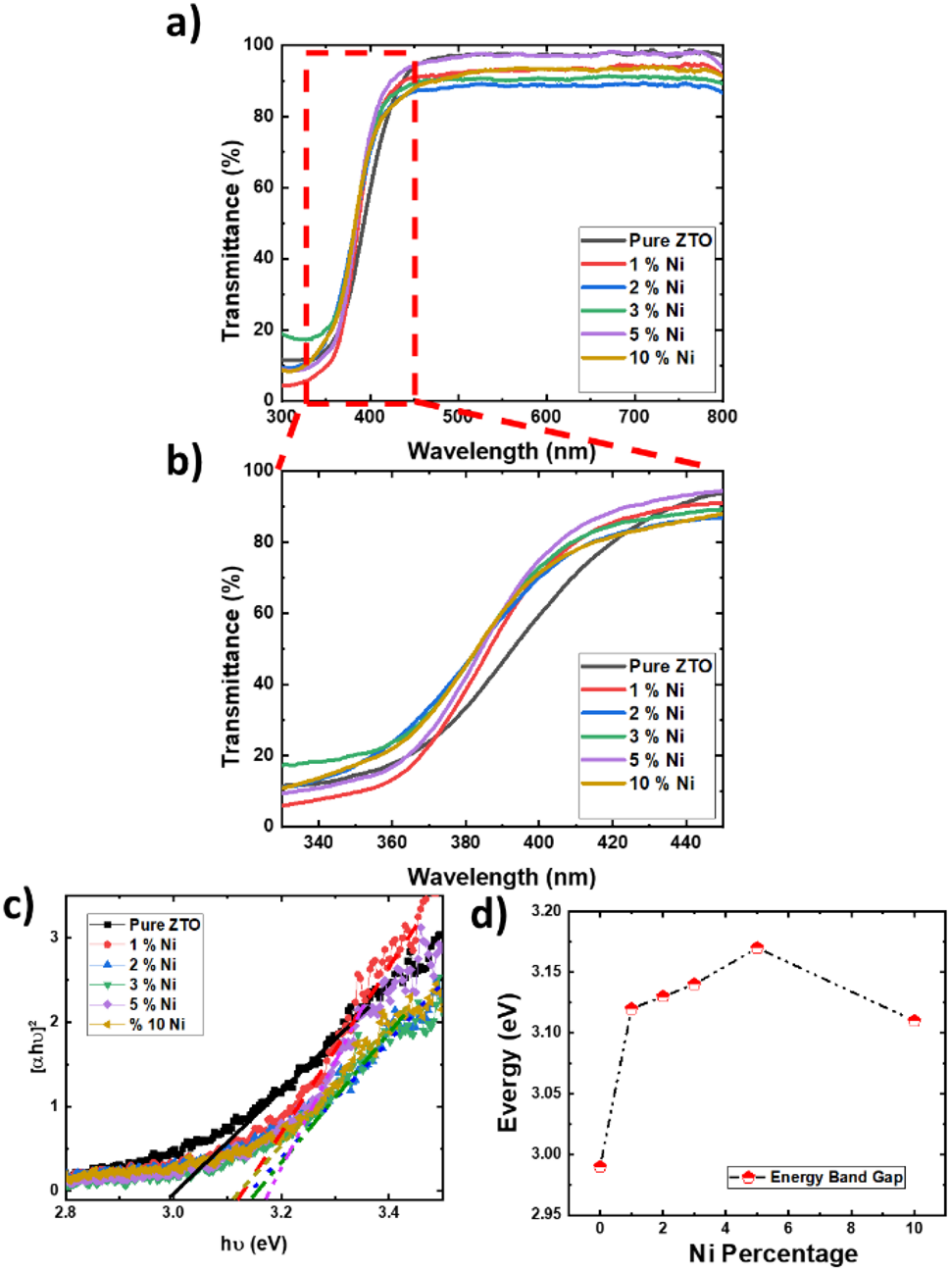
(**a,b**) UV-visible spectra of the pure and Ni-doped ZTO thin film. The (**c**) represents (αhv)^2^ versus photon energy.

To determine the optical band value of the obtained thin films, UV–visible spectroscopy was employed. Figure 4a, b display the transmittance spectra of pure and Ni-doped ZTO thin film with varying Ni concentrations. The absorption edge notably shifts towards shorter wavelengths, which correlates with the amount of Ni content present. To estimate the optical band gap, UV–visible spectra were initially analyzed using the Tauc method. The experimental optical band gaps were determined by plotting (αhv)^2^ as a function of photon energy and extrapolating the linear segment of the curve to intercept the photon energy axis and values of the optical band gap are illustrated in Fig. 4c, d.

It is evident from the results that the optical band gap of the Ni-doped ZTO thin film initially increases with increasing Ni concentration up to 5%, then decreases. The increase in band gap values results from the filling of the lowest states in the conduction band, a phenomenon attributed to the doping effect and well explained by the Burstein–Moss effect^51–53^. On the contrary, in heavily doped (over 10% doping), the decrease of band gap can be attributed the change in the nature and strength of the interaction potentials between donors and the host crystal^54,55^. This adjustment in the band gap value can improve the material's responsiveness for solar cell applications.

**Figure 5:**
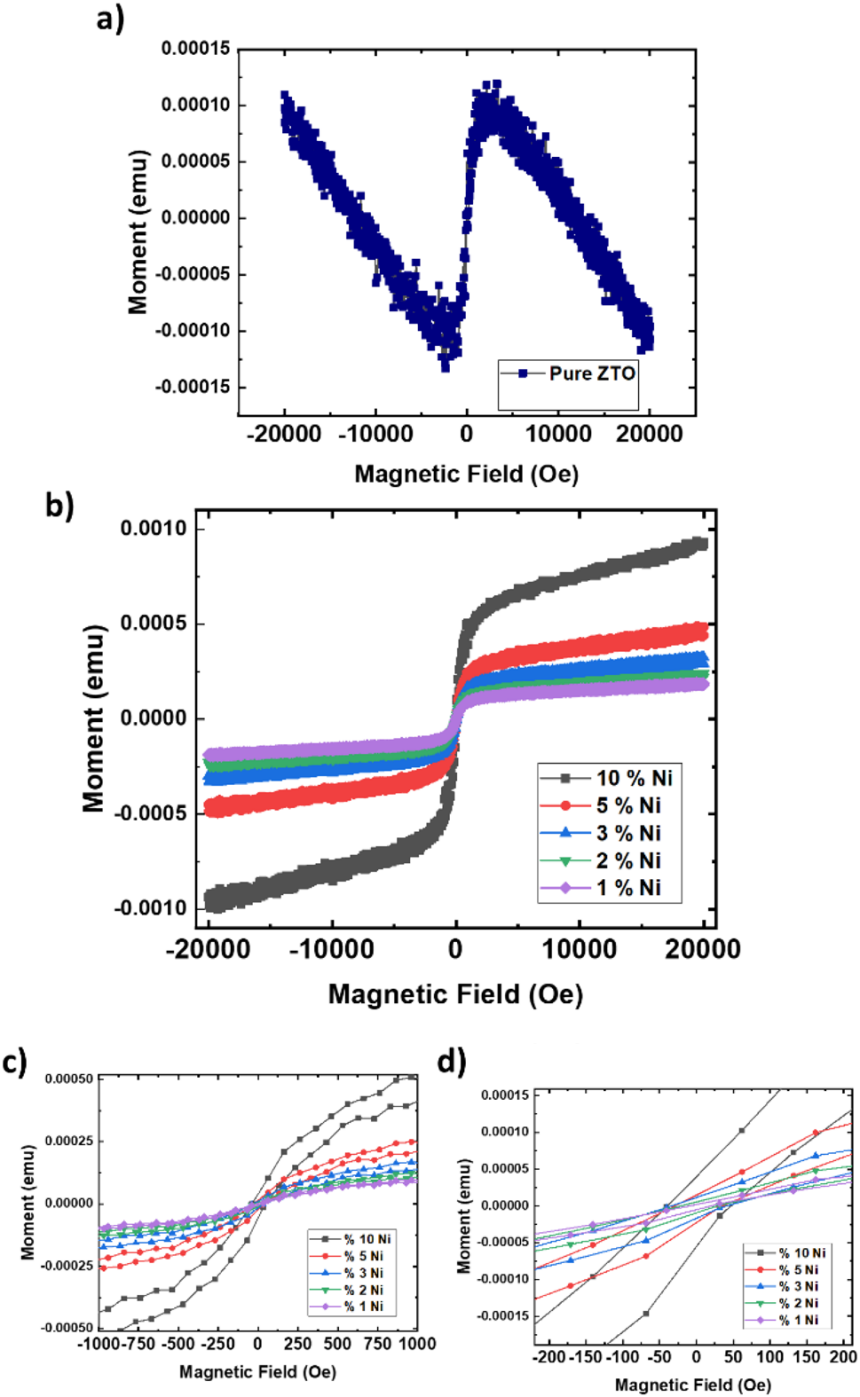
(**a**) Hysteresis loops of pure ZTO, (**b–d**) hysteresis loops of Ni-doped ZTO thin films at room temperature in different applied magnetic field scales.

The magnetic properties of the samples were examined using a vibrating sample magnetometer (VSM) at room temperature, with a maximum applied field of 20,000 G. In Fig. 5, magnetic hysteresis loops of both pure and Ni-doped ZTO thin films are presented. Pure zinc stannate thin film exhibited diamagnetic behavior, while the hysteresis loop for Ni-doped ZTO thin films displayed ferromagnetic property. The source of ferromagnetism in nonmagnetic semiconductors remains a topic of debate, with structural defects (such as oxygen vacancies, Zn, and Sn interstitials) and magnetic impurities often cited as explanations for magnetic ordering^56–60^. Studies have indicated that the emergence of ferromagnetism in non-magnetic oxides is contingent upon the appropriate level of dopant concentration and the formation of clusters on compound surfaces^56,61^. Additionally, the role of structural defects and magnetic impurities has been highlighted in elucidating magnetic behavior in nonmagnetic semiconductors. Therefore, our results can be explained with a super-exchange interaction between Ni ions and small magnetic dipoles located at the surface of the thin films. These dipoles further interact with their nearest neighbors within the material, potentially contributing to the observed ferromagnetism. Consequently, exchange interactions lead to the spin polarization of conductive electrons, ultimately resulting in ferromagnetism as the majority of Ni ions align in the same spin direction after successive long-range exchange interactions^20,34,37,38,62,63^.

The alteration in magnetic behavior may be attributed to Ni doping in ZTO, leading to the creation of structural defects (such as oxygen vacancies, Zn, and Sn interstitials) and magnetic impurities^30,34,63–65^. The obtained coercivity values for pure and Ni-doped zinc stannate thin film were measured at 512 G, 12 G, 25 G, 32 G, 37 G, and 51 G respectively. The change in coercivity field for doped ZTO underscores the influence of stoichiometry and occupancy at specific sites of Nickel. Furthermore, the magnetic properties, besides of coercivity field (H_c_), including remnant magnetization (M_r_) and saturation magnetization (M_s_), exhibited an increase with increasing Ni concentrations. From this results, having magnetic properties makes Ni doped ZTO as a potential material to use in spintronic application.

## Conclusion

In conclusion, this study successfully synthesized thin films of both pure and Ni-doped Zn2SnO4 using the sol–gel method. Analysis via XRD and SEM confirmed the formation of cubic crystal structures, with an intriguing observation of an increase in average crystallite size (from 1.57 to 6.70 nm) as Ni dopant concentration rose, without any discernible correlation. SEM images further illustrated the presence of cubic-shaped particles in all prepared samples. FTIR spectra revealed noticeable differences in absorption peaks compared to pure ZTO thin films, with peak intensities correlating with Ni dopant concentration. Moreover, the optical band gap energy values exhibited a trend of increasing with Ni concentrations up to 5% (from 2.99 to 3.17 eV), followed by a slight decrease at 10% Ni concentration (3.11 eV).

Of particular interest was the observation of magnetic properties in the diamagnetic ZTO thin film, which increased with the amount of Ni dopant. This highlights the potential of Ni doping to imbue magnetic characteristics into ZTO materials, thus broadening their applications in electronics and magnetism. Understanding the relationship between Ni dopant concentration and magnetic behavior provides valuable insights for the development of tailored functional materials. Further investigation into the mechanisms underlying the enhancement of magnetic properties in Ni-doped ZTO thin films is crucial to fully harness their potential in emerging technologies.

## Data Availability

The datasets used and/or analyzed during the current study available from the corresponding author on reasonable request.
